# Deep Neural Frameworks Improve the Accuracy of General Practitioners in the Classification of Pigmented Skin Lesions

**DOI:** 10.3390/diagnostics10110969

**Published:** 2020-11-18

**Authors:** Maximiliano Lucius, Jorge De All, José Antonio De All, Martín Belvisi, Luciana Radizza, Marisa Lanfranconi, Victoria Lorenzatti, Carlos M. Galmarini

**Affiliations:** 1 Topazium Artificial Intelligence, Paseo de la Castellana 40 Pl 8, 28046 Madrid, Spain; mlucius@topazium.com (M.L.); info@topazium.com (M.B.); 2 Sanatorio Otamendi, C1115AAB Buenos Aires, Argentina; jorgedeall@yahoo.com.ar (J.D.A.); jose.deall@medicus.com.ar (J.A.D.A.); rrpp@otamendi.com.ar (M.L.); victorialorenzatti@hotmail.com (V.L.); 3 Instituto de Obra Social de las Fuerzas Armadas, C1115AAB Buenos Aires, Argentina; lradizza@gmail.com

**Keywords:** artificial intelligence, dermatology, deep learning, skin diseases, melanoma

## Abstract

This study evaluated whether deep learning frameworks trained in large datasets can help non-dermatologist physicians improve their accuracy in categorizing the seven most common pigmented skin lesions. Open-source skin images were downloaded from the International Skin Imaging Collaboration (ISIC) archive. Different deep neural networks (DNNs) (*n* = 8) were trained based on a random dataset constituted of 8015 images. A test set of 2003 images was used to assess the classifiers’ performance at low (300 × 224 RGB) and high (600 × 450 RGB) image resolution and aggregated data (age, sex and lesion localization). We also organized two different contests to compare the DNN performance to that of general practitioners by means of unassisted image observation. Both at low and high image resolution, the DNN framework differentiated dermatological images with appreciable performance. In all cases, the accuracy was improved when adding clinical data to the framework. Finally, the least accurate DNN outperformed general practitioners. The physician’s accuracy was statistically improved when allowed to use the output of this algorithmic framework as guidance. DNNs are proven to be high performers as skin lesion classifiers and can improve general practitioner diagnosis accuracy in a routine clinical scenario.

## 1. Introduction

Diagnosis in dermatology is largely based on the visual inspection of a lesion on the suspicious skin area. Therefore, diagnostic ability and accuracy depend greatly on the experience and training of dermatologists or general practitioners, in areas where dermatological services are not readily available [1]. When dermatologists have no access to additional technical support, they have an approximately a 65–70% accuracy rate in melanoma diagnosis [2,3,4]. If the lesion is suspicious, the visual inspection is supplemented with different diagnostic tools (e.g., dermoscopy, confocal microscopy or optical coherence tomography) providing the ability to explore the skin in vivo, in depth and at a higher resolution [5,6]. However, access to these instruments remains limited due to time, logistical and cost concerns. Even when this technical support is feasible, dermatologists rarely achieve average rates greater than 85% [7,8]. The situation is even worse if we consider that there is a shortage of dermatologists whilst the diagnostic accuracy of non-expert clinicians is sensibly below than what is observed with dermatologists, reaching estimate rates between 20 and 40% [9,10,11,12,13]. Thus, new diagnostic tools assisting dermatologists or general practitioners to accurately diagnose skin lesions should be developed, evaluated and optimized.

Artificial intelligence (AI) is a computer science that involves creating sequences of data-related instructions that aim to reproduce human cognition [14]. The use of AI to assist physicians has been applied to various medical fields. In dermatology, image recognition using a set of algorithms called deep neural networks (DNNs) has proven to be of significant aid to physicians in the diagnosis of pigmented skin lesions. These algorithms achieve accuracies comparable to those of dermatologists [1,15,16,17,18]. In addition, Hekler et al. demonstrated that the combination of human and artificial intelligence is superior to the individual results of dermatologists or DNNs in isolation [19]. Similar results were observed in the case of non-pigmented skin lesions such as acne, rosacea, psoriasis, atopic dermatitis or impetigo. Thus, these technologies show tremendous promise to improve skin lesion diagnosis and may extend screening far beyond the clinical setting. However, many aspects of their use have yet to be elucidated and improved.

This study aimed to evaluate whether deep learning frameworks trained in large datasets can help non-dermatologist physicians improve their accuracy in categorizing the seven most common pigmented skin lesions representing more than 90% of the pigmented skin lesions. For this purpose, we (i) compared the accuracy of eight different DNNs in different training conditions such as the input of low and high image resolution and with or without clinical data to select the least performing DNNs; (ii) compare the accuracy of this DNN against non-dermatologist general practitioners; (iii) assess if these physicians improved their classification performance when using the framework as an assisting tool. Additionally, we developed an information maximizing generative adversarial network (infoGAN) to generate synthetic dermatological images [20].

## 2. Materials and Methods

### 2.1. Pigmented Skin Image Dataset

**Figure 1:**
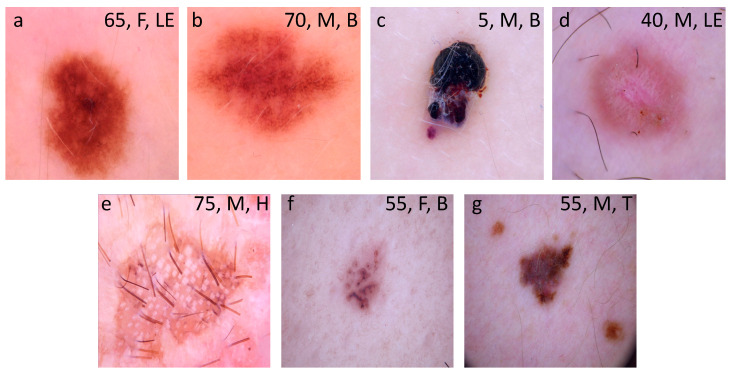
Examples of images downloaded from the HAM10000 dataset. These images are publicly available through the International Skin Imaging Collaboration (ISIC) archive and represent more than 95% of all pigmented lesions encountered during clinical practice (Tschandl P 2018). (**a**) Melanocytic nevus; (**b**) benign keratosis; (**c**) vascular lesion; (**d**) dermatofibroma; (**e**) intraepithelial carcinoma; (**f**) basal cell carcinoma; and (**g**) melanoma. Legends inside each image represents clinical data such as age, sex and localization associated to the image. F: female; M: male; LE: lower extremity; B: back; H: hand; T: trunk.

This study used images from the anonymous and annotated HAM10000 dataset publicly available through the International Skin Imaging Collaboration (ISIC) archive [21]. All downloaded images were selected using a random generator from the set of available images in the ISIC archive. We stochastically split the master set of 10,015 dermoscopic images into training (*n* = 8313; 83%) and test (*n* = 1702; 17%) datasets that were completely disjoint. Images included a representative collection of all-important diagnostic categories across the seven different types of pigmented lesions as detailed in Tschandl et al. [21]. These included melanocytic nevus, vascular skin lesions (including cherry angiomas, angiokeratomas, pyogenic granulomas and hemorrhages), benign keratoses (including seborrheic keratoses, solar lentigo and lichen-planus-like kertoses), dermatofibroma, intraepithelial carcinoma (including actinic keratoses and Bowen’s disease), basal cell carcinoma and melanoma. Examples of images of each lesion type are depicted in Figure 1. The final composition of each dataset is shown in Table 1.

### 2.2. Deep Neural Networks

We evaluated eight different DNNs, each characterized by a specific architecture. VGG16 and VGG19 contain 16 and 19 convolutional layers, respectively, with very small receptive fields, five max-pooling layers of size for carrying out spatial pooling, followed by three fully connected layers, with the final layer as the soft-max layer [22]. Rectification nonlinearity (ReLu) activation is applied to all hidden layers. The model also uses dropout regularization in the fully connected layers. ResNet34 is a 34-layer residual network while ResNet50 and ResNet101 are 50- and 101-layers deep, respectively. The architecture of all these DNNs is similar to the one found in VGG consisting mostly of 3 × 3 filters, however, instead, shortcut connections are inserted resulting into a residual network. SEResNet50 architecture is based on ResNet. A squeeze-and-excitation block is applied at the end of each non-identity branch of residual block [23]. Differently, and instead of increasing its size by adding more or deeper layers, EfficientNetB5 scales up the network width, depth and resolution with a set of fixed scaling coefficients [24]. Finally, MobileNet uses depth-wise separable convolutions which significantly reduce the number of parameters when compared to a network based on standard convolutions and the same depth across the structure in the networks [25]. The framework is 54 layers deep.

For each DNN, the initial weights of all layers of the network were set up after pretraining with ImageNet. To assess both the performance of the algorithm and the enhanced training techniques as accurately as possible, we retrained each DNN a total of five times (folds), and each training run consisted of 90 epochs. Training using the curated image patches took approximately 6 h to complete, 45k iterations on a 4 GeForce GTX 1080 GPU configuration. Training accuracy for the curated patches reached maximum accuracy (100%) at around epoch 32, whereas the pretrained model only began to converge around epoch 25. All these DNNs were trained and tested with two different image input size: 300 × 224 RGB and 600 × 450 RGB. The low-resolution images were obtained by cropping, distorting and linear resizing the original high-resolution images. DNNs were also trained and tested without or with the clinical features (sex, age and location of the lesion) associated to every image in the HAM10000 database.

### 2.3. Image Preprocessing

When a deep convolutional neural network overfits, it works extremely well on training data but poorly on data it has never seen before. This is especially important in the field of dermatology because of the variability that exists in the images that the neural network will be analyzing. Two steps were taken to reduce overfitting. First, a dropout layer was added and set to 0.5. This results in 50% of the neurons to be randomly turned off during the training process and therefore reduce the likelihood of overfitting. The second step taken to reduce overfitting was to use data augmentation. In data augmentation, the images are modified to account for some of the variability that exists in image taking. To account for the grid location, the size of the dermatological manifestation and the angle of the image, the training images fed into the model were altered using zoom (25% probability to be increased between −1 and 2%, rotated between −6º and 6º and vertical/horizontal transfer from −2 to 2%), rotation (25% probability of being randomly rotated 90º clockwise), transposing (15% probability of making a random axial symmetry in one of its diagonals) and horizontal and vertical flipping randomly (50% probability of making a horizontal or vertical “mirror” or both) or following optical parameters from the different types of phone cameras such as shear and brightness (30% probability of contrast modification between −8 and 8%) or optical (80% probability of being distorted with a range between −6 and 6%) or grid (75% probability at step 4 with a range of −28–28%) distortions. These modifications were applied to 66% of the input images. The model was run once without data augmentation with dropout and once with data augmentation including drop out in both instances.

### 2.4. Generation of Synthetic Pigmented Skin Lesion Images Using an infoGAN

Generative adversarial networks (GANs) are a type of generative model that attempt to synthesize novel data that are indistinguishable from the training data [26]. They consist of two neural networks, locked in competition: a generator that captures the data distribution and creates synthesized data (e.g., an image), and a discriminator that estimates the probability that a sample came from the training data rather than from the generator. The two networks are sealed in a zero-sum game, where the success of one corresponds to the failure of the other. The training procedure for the generator is to maximize the probability of the discriminator making an error [27]. Thus, this framework is based on a value function that one model seeks to maximize and the other seeks to minimize. Since the two networks are differentiable, the system generates a gradient that can be used to steer both networks to the right direction.

We trained an information maximizing generative adversarial network (infoGAN) composed of a generator and a discriminator, on all high resolution images of the HAM10000 dataset [20]. The infoGAN was adapted to the progressive architecture of the model by splitting the structured code into parts and feeding each part to the network by conditioning activation in the corresponding block of the generator (Supplementary Figure S1). To prevent detrimental competition between the discriminator and generator, and to achieve convergence in an efficient way, we followed the recommendations detailed in Chen et al. [20]. Briefly, the discriminator and generator were composed in eight progressive blocks with an input/output of spatial resolution of 4 × 4 in the initial step up to 512 × 512 in step 7. The Batch size has gone dynamically from 128 in step 0 to 2 in the last step. Both the generator and discriminator were optimized using Adam with an initial learning rate of 0.0075 and exponential decay of 0.99 and a Wasserstein function with gradient penalty. The training has progressed in phases of progressive increased resolution. More specifically, the model was capable of generating high resolution images with isolated semantic features controlled by a set of real valued variables. Color, age, sex, localization and type of lesion were the most important semantic features discovered during the training in a completely unsupervised fashion without human input. After training, the generator produced novel images, similar to those in the dataset.

### 2.5. Contests among General Practitioners

To compare the accuracy of DNNs with non-dermatologist practitioners, we conducted two different challenges. The first aimed to establish the accuracy of general practitioners in classifying images from the HAM10000 dataset without time constraint. For this purpose, a group of 22 general practitioners from any given center in Buenos Aires (Argentina) were given access to 163 images of the different skin pigmented lesions through an anonymous website specifically created for this purpose. Physicians could enter and exit the website without limitation. Alongside the image, recorded factors such as the age, sex and localization of the lesion were shown. All physicians were asked to classify every image within the seven different diagnostics. No incentives were offered for participation. To ensure fair comparisons between the results determined by general practitioners and those determined by the DNNs, the same 162 images were run with the DNN framework which had the worse accuracy metrics in low-resolution and without aggregated clinical features of the eight DNNs tested.

To determine if physicians could benefit from access to the algorithmic tool during the own classification task, a second evaluation was conducted. A group of 19 general practitioners that voluntarily accepted to participate in the study was first asked to assess 35 images in a simulated exercise with time constraints (physicians had 45 s to classify every image). In a second step, physicians had access to the predictions of the same algorithmic framework used during the first challenge, the same group having classified each image based on both their criteria and the algorithmic output. For this task, a new set of 35 images was shown with the same time constraints. In both contests, the ethics committee waived ethical approval owing to the use of anonymized dermatologic images obtained from the publicly available HAM10000 dataset.

### 2.6. Statistical Analysis

After the model had been trained, a test step was performed in which 1702 images of the seven dermatological manifestations were used as input and the results were statistically analyzed. A confusion matrix was constructed based on comparing the frameworks’ prediction with each of the actual labels. All analyses were performed and programmed via a Jupyter notebook in Python. Sensitivity, specificity, geometric mean, accuracy and error rate were calculated for each dermatological manifestation [28]. Sensitivity or true positive rate (TPR) represented the positive and correctly classified samples to the total number of positive samples. The specificity or true negative rate (TNR) was estimated as the ratio of the correctly classified negative samples to the total number of negative samples. Geometric means were calculated by using the product of TPR and TNR. Accuracy was defined as the ratio between the correctly classified samples to the total number of samples [28]. We also calculated the error rate as the complement of accuracy. All these measures are suitable to evaluate the classification performance based on imbalanced data as found in the HAM10000 database. All metric results were calculated with respect to the class labels documented in the HAM10000 database archive. A summary diagram is depicted on Supplementary Figure S2.

## 3. Results

### 3.1. Classification Metrics across Eight Different DNNs

The results of global accuracy and error rate for each DNN for the classification of seven pigmented skin lesions at low image resolution (300 × 224 RGB) are shown in Table 2. The average global accuracy for the eight DNNs reached 76.30% ± 2.79, ranging from 74.05% (EfficientNetB5) to 82.47% (MobileNet). As shown in Supplementary Table S1, the TPR, TNR and geometric mean for each disease subtype varied according to the tested DNN. Almost all DNNs showed the highest TPR for melanocytic nevi classification when compared to the other pigmented lesions, with observed geometric mean values equal or lower than 0.65; interestingly, VGG16, VGG19 and MobileNet also showed high TPR for vascular lesion classification (geometric mean values lower than 0.65). In the case of melanoma and benign keratosis classification, all of them showed a TPR of approximately 0.5, with geometric mean values around 0.75. Likewise, basal cell carcinoma classification showed a TPR of approximately 0.5 and geometric mean values higher than 0.75 using ResNet50, ResNet101, SEResNet50 and EfficientNetB5. Similar results were observed for SEResNet5 and MobileNet and intraepithelial carcinoma classification.

Using higher image resolution (600 × 450 RGB) improved the global accuracies across all DNNs (Table 2). The global accuracy average for the eight DNNs tested was 77.76 ± 2.77, ranging from 75.50 (EfficientNetB5) to 83.88% (MobileNet). Although higher than the mean average observed with low-resolution images, the difference was not statistically significant (*p* = 0.07; Mann–Whitney U test). The TPR, TNR and geometric mean values for each disease subtype are detailed in Supplementary Table S2. The highest TPR was observed for melanocytic nevi across all DNNs, and in the case of vascular lesions using VGG16 and MobileNet. On the contrary, the lowest TPR was observed for dermatofibrosis and intraepithelial carcinoma with ResNet34, ResNet50, SEResnet50 and EfficientNetB5. When compared with the low-resolution image parallel cases, the TPR, TNR and geometric mean values were quite similar for most of the disease subtypes. However, a drastic improvement of TPR was observed for dermatofibrosis using ResNet34 (from 0.23 to 0.34), ResNet50 (from 0.29 to 0.55), ResNet101 (from 0.26 to 0.42), VGG16 (from 0.17 to 0.4), VGG19 (from 0.26 to 0.40) and MobileNet (from 0.39 to 0.5). Similar TPR improvements were observed for the intraepithelial carcinoma classification using VGG19 (from 0.26 to 0.40). The average cascade framework runtime of the high-resolution classification model was 21.46+/−2.3 milliseconds per image, whereas the low-resolution model required only 18.6+/−1.22 milliseconds per image. Altogether, these results indicate that the tested DNNs can classify seven different types of pigmented skin lesions with accuracies higher than 0.7. No major differences in runtime were observed between the cascade framework input with low- or high resolution.

### 3.2. Classification Metrics of Different DNNs Aggregating Image and Clinical Features

We then investigated if adding clinical features to the analysis could improve the classification accuracy of each DNN. As the HAM10000 dataset provides the sex, age and localization of the skin lesion associated to every image, we gathered these clinical features and aggregated them with the corresponding image to be used as input for each of the tested DNNs. Results are shown in Table 3. For low-resolution images, the addition of clinical data improved the global performance of all DNNs but MobileNet. The average global accuracy for the eight DNNs was 78.86% ± 1.81, ranging from 75.73 (EfficientNetB5) to 81.24% (MobileNet). This represented a statistically significant increase in comparison to the global accuracy of DNNs tested with low-resolution images without aggregated clinical features (*p* = 0.004; Mann–Whitney U test). The highest increase was observed for VGG19 raising from 74.21% to 79.43 (5.22%). As shown in Supplementary Table S3, classification improvements were observed in almost all pigmented skin lesions for all DNNs. The highest marginal increases in accuracy were observed for dermatofibrosis where the TPR values for ResNet 50, SEResNet50, VGG16, EfficientNetB5 and MobileNet were raised by 15, 51, 22, 28 and 46%, respectively. Similarly, in the case of the intraepithelial carcinoma condition, TPR values increased 13 and 26% for SEResnet50 and VGG19, respectively, and for basal cell carcinoma classification, increased by 11, 14 and 12% for ResNet50, VGG19 and EfficientNetB5, respectively. For vascular lesions, the TPR values increased by 34, 14 and 29% for ResNet101, VGG19 and EfficientNetB5, respectively. Finally, adding clinical features to skin images also improved the melanoma condition accuracy for VGG16 (from 0.47 to 0.61) and VGG19 (from 0.44 to 0.58). Of note, a decreased TPR was observed for ResNet101 in the classification of dermatofibrosis (from 0.26 to 0.16) and intraepithelial carcinoma (from 0.37 to 0.29), and in melanoma for MobileNet (from 0.90 to 0.72).

For high-resolution images, the average global accuracy also increased when clinical features were added to the model (Table 3). The average global accuracy was 80.22% ± 2.30, ranging from 77.14 (EfficientNetB5) to 84.73% (MobileNet). This represented a statistically significant increase in comparison to the global accuracy of DNNs tested with high-resolution images without aggregated clinical features (*p* = 0.02; Mann–Whitney U test). This was particularly evident in the case of ResNet50 performance with an increase of 4.75%. The TPR, TNR and geometric mean values are shown in Supplementary Table S4. Major TPR improvements were observed for dermatofibrosis with ResNet34 (26%), ResNet101 (20%), VGG19 (17%) and EfficientNetB5 (28%). TPR increases were also observed for basal cell carcinoma with ResNet50 (12%) and EfficientNetB5 (13%), for vascular lesions with ResNet101 (37%) and EfficientNetB5 (28%), for melanoma with VGG16 (14%), VGG19 (32%) and MobileNet (16%) and intraepithelial carcinoma with VGG16 (24%). Interestingly, the TPR of ResNet50 and SEResNet50 were reduced for dermatofibrosis from 0.55 to 0.20 and from 0.44 to 0.29, respectively. When compared to the DNNs’ performance with low-resolution images and clinical features, no major differences were observed (*p* = 0.21; Mann–Whitney U test). Of interest, for dermatofibrosis classification, the TPR increased for ResNet34 (35%), ResNet101 (50%), VGG16 (50%) and VGG19 (24%); the exception was ResNet50 with a TPR reduction from 0.44 to 0.2. Altogether, these results indicate that the addition of information related to sex, age and localization of the lesion improves the accuracy of DNNs.

### 3.3. Performance across Synthetic Pigmented-Skin Lesion Images

**Figure 2:**
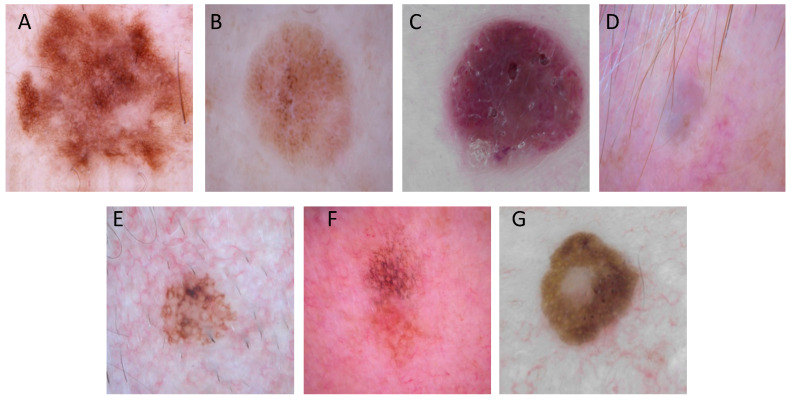
Examples of synthetic images generated with the information maximizing generative adversarial network (infoGAN): (**A**) melanocytic nevus; (**B**) benign keratosis; (**C**) vascular lesion; (**D**) dermatofibroma; (**E**) intraepithelial carcinoma; (**F**) basal cell carcinoma; and (**G**) melanoma.

We applied an infoGAN to generate synthetic images from the HAM10000 dataset. As shown in Figure 2, the synthetic images seemed realistic and diverse. We then calculated the global accuracy for EfficientNetB5 for the classification of seven pigmented skin lesions. Of the 40 synthetic images analyzed, the network made a single error, so the certainty index was 97.5%; however, this value lacks any relevance since the GAN used for pseudo-labeling deliberately increases the definition limit of each class, inducing an improvement in the certainty of the classifier. Altogether, these data indicate that the synthetic samples are highly realistic and can be used as inputs to train DNNs on pigmented skin lesion classification.

### 3.4. Performance across General Practitioners with and without Assistance from DNNs Output

In the first challenge, 22 general practitioners were asked to classify 162 images without any time constraint. The mean global accuracy and mean error rate were 27.74 and 72.26%, respectively. These results were similar to those previously published for non-dermatologists [9,10]. The best TPR (0.79) was obtained for the melanocytic nevi while the worse metrics were observed for vascular lesions (0.02), dermatofibrosis (0.01) and intraepithelial carcinoma (0.07) (Supplementary Table S5). As EfficientNetB5 was slightly less accurate than the other tested DNNs, we decided to use this framework as a comparator (see Table 1). In the same dataset, this DNN had a mean global accuracy of 78.40% and mean error rate 21.60% (Table 4). Compared to physicians, this was a relevant and significant difference as EfficientNetB5 showed a higher TPR in all disease subtypes (Supplementary Table S5).

In the second challenge, 19 general practitioners were asked to classify 35 images with a time constraint of 45 s per image. The global accuracy for this classification was 17.29% (Table 4). In the same dataset, EfficientNetB5 achieved a global accuracy of 77.14%, significantly outperforming physicians. When general practitioners were given the opportunity to access the output of EfficientNetB5 per image, the global accuracy increased to 42.42%. This represented an increase of 25.13%. This result also indicated that, in some cases, physicians did not follow the recommendation of the DNN. Of note, the access of physicians to DNN prediction increased TPR for basal cell carcinoma (from 0.10 to 0.54) and melanoma (from 0.08 to 0.35) (Supplementary Table S6). In contrast, a small decrease in TPR for benign keratosis was observed (see Supplementary Table S6).

Altogether, these results show that DNNs have the capability to classify seven different pigmented skin lesions with a level of competence higher to that of the general practitioners participating in these challenges. The access of DNN output by physicians improves their ability to classify pigmented skin lesions, particularly basal cell carcinoma and melanoma.

## 4. Discussion

Our results demonstrate that deep learning frameworks trained on large, open source image datasets can help non-dermatologist physicians improve their accuracy to categorize the seven most frequent pigmented skin lesions. Additionally, we showed that image resolution does not affect the performance of eight different DNNs. Instead, the aggregation of clinical features (age, sex and lesion localization) significantly increases DNN performance with both low-resolution and with high-resolution image inputs. The use of artificial intelligence as a diagnostic aid is a growing trend in dermatology. A digital automated skin assistance tool provides undeniable help for dermatologists and general practitioners to reduce the morbidity and mortality linked to dermatological diseases by favoring early diagnosis and by the avoidance of unnecessary procedures. The advent of deep/machine learning algorithms has made the automated classification of cutaneous lesions an achievable target milestone [29].

Different dermatologic studies have reported early success in the classification of pigmented skin lesions from both clinical and dermoscopic images with a level of accuracy comparable to that of dermatologists. Esteva et al. were among the first ones to describe a DNN that performed as well as dermatologists when identifying images with malignant lesions [15]. The authors used a GoogleNet Inception v3 architecture that was pre-trained on approximately 1.28 million images. Then, they used 129,450 skin images of 2032 different diseases to train and ultimately validate the system using two classes (benign/malignant). The model was compared to the performance of 21 dermatologists using a test set of 135 biopsy-proven lesion clinical and dermoscopic images. The performance of this binary classification method was on par with that of all of the dermatologists who participated. Haenssle et al. presented a very similar approach to Esteva et al. [1]. They compared the diagnostic performance of 58 dermatologists with a GoogleNet Inception v3 model that was adapted for skin lesion classification with transfer learning, whereby the weights were fine-tuned in all layers. The analysis was limited to dermoscopic images of melanoma vs. benign nevi. In the test dataset of 300 biopsy-proven images, the accuracy of the DNN compared favorably with the one by dermatologists. Likewise, Han et al. presented a ResNet152 classifier for 12 different skin diseases based on clinical images that performed comparably to the performance of 16 dermatologists [16]. Fujisawa et al. used a dataset of 4867 clinical images to train a DNN to differentiate 14 different clinical conditions that included both malignant and benign conditions [30]. The machine’s performance was then compared against that of 13 dermatologists and nine dermatology trainees and tested on 1142 images distinct to those used for training. The DNN outperformed the dermatologists across every field. Additionally, a set of other recent studies also reached dermatologist-level skin cancer classification by using DNNs [31,32,33,34,35,36,37].

In contrast to all these previously mentioned publications comparing the performance across different DNN configurations to the one by dermatologists, our study was carried out with non-dermatologist practitioners. Our results show that the tested frameworks classify pigmented skin lesions much better than the general practitioners that participated in this study. Our results are similar to those recently published by Tschandl et al. [38]. These authors have matched a set of DNNs with human readers for the diagnosis of seven clinically relevant types of pigmented skin lesions analyzed in our study using the HAM10000 dataset. From the 511 human readers involved in their study, 83 were general practitioners. The authors showed that the top three DNNs outperformed physicians with respect to most outcome measures. However, human metrics were only disclosed for dermatology experts, and thus, we cannot compare our metrics to them. Although promising, our results should be analyzed within the context as they are derived from a set of pre-existing images and not from a real-life patient observation. Indeed, general practitioners are not trained to diagnose over an image, particularly if they have just a few seconds to decide. Moreover, in a real-world situation, they would consider other clinical features besides a skin image and given complementary data; they would be evaluating the patient as a whole, not just a skin lesion. In spite of this, our results showing that a physician’s access to a standard individual DNN output improved their ability to classify pigmented skin lesions are encouraging. Moreover, this assistance improved the positive classification of basal cell carcinomas, one of the most common of all types of cancer, and the most dangerous melanoma.

At a more technical level, our results are in agreement with various other publications that have also demonstrated the capacity of multiple DNN constructs to classify clinical or dermoscopic skin images [17,39,40,41,42,43,44,45]. Some of these classifiers have been translated onto online platforms and smartphone applications for use by dermatologists or individuals in the community setting (e.g., modelderm, MoleMapper, MoleAnalyzer Pro) [46]. Most of these published works use non-public archives [1,15]. This makes it very difficult to reproduce the results and compare the performance of published classifiers against each other. Thus, we decided to compare the performance of eight different individual DNNs using a unique and public dataset, namely the HAM10000. Among other things, we showed that the quality of the input images marginally affects the performance of a classification task for a given DNN, as similar achievements have been made with both low- and high-resolution image as input. According to our results, the higher the resolution of the image, the better the performance of a given DNN is; however, the improvements were not so evidently compared to those observed with the addition of a few clinical features to the analysis. Indeed, the algorithms trained with low-resolution images and aggregated clinical features achieve levels of precision similar to those obtained with better resolution images without clinical features. This is in line with a recent publication that showed that adding clinical information to skin lesion images improves the diagnostic accuracy of dermatologists [1]. This result is significant as it implies that adding clinical features is more important than the resolution of the input image, the best situation being the combination of high-resolution images and clinical features. From a clinical perspective, it is important to note that other authors have used other complex mathematical techniques to improve the algorithm’s performance, such as dropout, data augmentation and batch normalization [47,48,49]. Data augmentation along with a larger database including both higher resolution images and clinical data such as symptoms and the localization of such an image could sensibly improve the image with higher probability. Of note, MobileNet showed the consistent best performance while one of the latest developed DNN (EfficientNetB5) gave surprisingly consistently the worst performance. This seems counterintuitive as MobileNet is considered a simplified version of the other deep learning networks. The MobileNet model is based on depthwise separable convolutions which is a form of factorized convolutions which factorize a standard convolution into a depthwise convolution and a 1 × 1 convolution called a pointwise convolution [25]. This factorization has the effect of drastically reducing the computation and model size. We believe that the best performance of MobileNet could be due to the fact that this network extracts the optimal number and the most relevant features better compared to other neural networks. Indeed, a recent study has described that the optimal number of extracted features on dermoscopic images seems to vary depending on the method’s goal, and that extracting a large number of features can lead to a loss of model robustness [50]. Similarly, although described as a particular performant deep network, EfficientNetB5 could be showing worse performance in extracting the optimal number and more relevant features for the dermatological images used in this study.

In this work, we used the HAM10000 dataset from the ISIC archive (https://isic-archive.com/) (access date: 1 November 2019) [21]. As members of the scientific community, we are truly grateful to the researchers who have generated this dataset and we acknowledge the enormous effort invested in it. Because of permissive licensing, well-structured availability, and large size, it is currently the standard source for dermoscopic image analysis research. Although other open skin lesions datasets containing clinical and dermoscopic images are available, these are not as large as the HAM10000, leaving it as the only public one that can be used for the training and validation of new algorithms [51]. We set out to solve this problem by generating synthetic images using an infoGAN. This framework consists of a generator network that tries to produce realistic looking images, while a discriminator network aims to classify well between samples coming from real training data and fake samples generated by the generator [26,27]. Our results show that the synthetic skin images can be used as input images by DNNs, similar to that observed with real images of the HAM10000 dataset. This indicates that this synthetic dataset can be used to train and test different algorithmic frameworks, overcoming the lack of skin image databases representing the diversity of skin types observed in the real world. As described in Tschandl et al., however, the HAM10000 dataset presents some flaws [21]. First, it is biased towards melanocytic lesions (12,893 of 13,786 images are nevi or melanomas). Likewise, although it is an excellent curated image dataset, it is mostly composed of skin images of a mostly fair-skinned Caucasian population thus making difficult to extrapolate the results to other racial groups. Indeed, it was recently reported that a DNN trained on an image dataset composed mainly of Caucasian population skin images (Fitzpatrick 1 and 2 skin types) could not be extrapolated to African-black skin color patients [52]. In that study, the DNN’s accuracy was as low as 17%. Other open skin lesions datasets containing clinical and dermoscopic images from non-Caucasian human skin types are available, however, these are not as large as the HAM10000 or are not publicly available [51]. To tackle this problem, and based on the results shown in this work, we are generating synthetic images representing the different pigmented lesions of the HAM10000 dataset on the six human skin types according to the Fitzpatrick scale using an infoGAN [20].

In terms of the limitations within our study, firstly, the algorithms were bound to only seven different disease classes which does not reflect clinical reality as many more options should be taken into account when diagnosing [53]. As a consequence, the use of these classification algorithms should be regarded as an assisting tool for dermatologists or general practitioners that may improve accuracy within a limited scope but not as a replacement for independent diagnoses qualified by a supervising physician. Secondly, deep learning models are powerful “black box” models which remain relatively uninterpretable compared to the statistical methods used in medical practice [54]. Computer vision models combine pixel-based visual information in a highly intricate way, making it difficult to link the model output back to the visual input. A third limitation is that although the test dataset was disjunct from the training dataset, all the images belonged to the same database (HAM10000), raising concerns about their ability to generalize on a truly external test set coming from a different image bank. It is known that the efficacy of DNNs varies based on the set of images with which they are trained. Each model may have different sensitivities and specificities and may be subject to a unique set of biases and shortcomings in prediction introduced by the image training set. In a recent study, a binary-classification DNN for melanocytic nevus vs. melanoma, trained on ISIC images, showed good performance on an ISIC test dataset but performed badly on an external test dataset from the PH2 dermoscopic image source [1,51]. Using just 100 images from the external database for fine-tuning the DNN sufficed to completely restore the original performance. Another important issue is related to the artifacts observed in clinical or dermoscopic images, such as surgical skin markings, dark corners, gel bubbles, superimposed color charts, overlayed rulers, and occluding hair that can affect image classification by automated algorithms [55]. Various methods have been reported for the removal of such artifacts and strategies for preprocessing images were described to improve the classification outcomes of DNNs [55,56,57,58]. Finally, a major point of weakness of our study was the lack of comparison with traditional, hand-designed image descriptors [50,59,60]. Although it has already been published that the effectiveness of DNNs outperforms those of hand-crafted descriptors, these methods were shown to be better at discriminating stationary textures under steady imaging conditions and proved more robust than DNN-based features to, for example, image rotation [61]. Moreover, the concatenation of handcrafted features (shape, skeleton, color, and texture) and features extracted from the most powerful deep learning architectures followed by classification using for standard classifiers (e.g., support vector machines) was shown to offer high classification performance [62]. Future efforts would be directed to confirm if our approach offers any advantages compared to the hand-designed methods.

## 5. Conclusions

In conclusion, our findings show that deep learning algorithms can successfully assist non-dermatologist physicians in potentiating their classification performance across seven different pigmented skin lesions. Moreover, this technology would help primary care physicians in the decision-making process on which patients are at highest risk for skin cancer, with subsequent referral to dermatology for total body skin examination. These models could be easily implemented in a mobile app, on a website, or even integrated into an electronic medical record system enabling fast and cheap access skin screenings, even outside the hospital. Future research should carefully validate our results using other image datasets containing patients across a blend of different ages and ethnicities, including additional cutaneous lesions and skin color types. Ultimately, automated diagnostic systems based on DNNs will allow clinicians to enhance patient care by means of improving their classification skills outside of their field of expertise.

## Figures and Tables

**Table 1:** Description of the training and test datasets.

Class	Training Set (*n*)	Test Set (*n*)
Melanocytic nevi	5565	1140
Benign keratoses ^1^	912	186
Vascular lesions ^2^	118	24
Dermatofibroma	96	20
Intraepithelial carcinoma ^3^	271	56
Basal cell carcinoma	427	87
Melanoma	924	189
Total	8313	1702

^1^ includes seborrheic keratoses, solar lentigo and lichen-planus like keratoses. ^2^ includes cherry angiomas, angiokeratomas, pyogenic granulomas and hemorrhage. ^3^ includes actinic keratoses and intraepithelial carcinoma (Bowen’s disease).

**Table 2:** Classification metrics of HAM10000 images at two different resolutions and without aggregated clinical features using eight different DNNs.

	Low-Resolution Images ^1^		High-Resolution Images	
DNN ^2^	Accuracy ^3^	Error Rate	Accuracy	Error Rate
ResNet34	75.32	24.68	76.73	23.27
ResNet50	74.56	25.44	75.97	24.03
ResNet101	75.62	24.38	77.02	22.98
SEResNet50	77.82	22.22	79.13	20.87
VGG16	76.85	23.15	78.25	21.75
VGG19	74.21	25.79	75.62	24.38
EfficientNetB5	74.05	25.91	75.50	24.5
MobileNet	82.47	17.53	83.88	16.12

^1^ low-resolution image: 300 × 224 RGB; high-resolution image: 300 × 224 RGB. ^2^ Deep neural network. ^3^ accuracy and error rates are expressed as percentages.

**Table 3:** Classification metrics of HAM10000 low- and high-resolution images with aggregated clinical data using eight different DNNs.

	Low-Resolution Images ^1^		High-Resolution Images	
DNN ^2^	Accuracy ^3^	Error Rate	Accuracy	Error Rate
ResNet34	77.43	22.57	78.84	21.16
ResNet50	79.31	20.69	80.72	19.28
ResNet101	77.55	22.45	78.96	21.04
SEResNet50	80.01	19.99	80.72	19.28
VGG16	80.25	19.75	81.65	18.35
VGG19	79.43	20.57	79.02	20.98
EfficientNetB5	75.73	24.27	77.14	22.86
MobileNet	81.24	18.76	84.73	15.27

^1^ low-resolution image: 300 × 224 RGB; high-resolution image: 300 × 224 RGB. ^2^ Deep neural network; ^3^ accuracy and error rates are expressed as percentages.

**Table 4:** Classification metrics of non-dermatologists, general practitioners with or without use of the algorithmic platform and with time constraints.

Condition	Accuracy ^1^	Error Rate
EfficientNetB5	77.14	22.86
GPs ^2^	17.29	82.71
GPs + AI	42.43	57.57

^1^ accuracy and error rate are expressed as percentages. ^2^ GPs: general practitioners; AI: artificial intelligence.

## Supplementary Materials

The following are available online at https://www.mdpi.com/2075-4418/10/11/969/s1, Figure S1: Representation of a generic block; Figure S2: Summary diagram of the methodology; Table S1: Classification metrics for each skin lesion subset of the HAM10000 images using eight different CNNs and low-resolution images without associated clinical features, Table S2: Classification metrics for each skin lesion subset of the HAM10000 images using eight different CNNs and high-resolution images without associated clinical features, Table S3: Classification metrics for each skin lesion subset of the HAM10000 using eight different CNNs and low-resolution images with aggregated clinical data, Table S4: Classification metrics for each skin lesion subset of the HAM10000 using eight different CNNs and high-resolution images with aggregated clinical data, Table S5: Classification metrics for each skin lesion in 163 images of the HAM10000 database by non-dermatologist, general practitioners without access to algorithmic outputs and no time constraint, Table S6: Classification metrics for each skin lesion in 70 images of the HAM10000 database by non-dermatologist, general practitioners without (*n* = 35) or with access (*n* = 35) to algorithmic outputs and time constraint of 45 sec/image.
