# Supplementary material for: Deep Neural Frameworks Improve the Accuracy of General Practitioners in the Classification of Pigmented Skin Lesions
Source: Diagnostics (Basel). 2020 Nov 18;10(11):969. doi: 10.3390/diagnostics10110969 (PMC7698907; doi:10.3390/diagnostics10110969)
Supplement: Supplementary file 1 [file diagnostics-10-00969-s001.zip › New folder/diagnostics-989302-supplementary-FIGURE.pdf]

# Supplementary Material

## Supplementary Figure 1. Representation of a generic block

d = 3 … 9
**Inp**:  shape=2**(d-1),2**(d-1), n_channels[d-1]
**upsample**: shape= 2**(d),2**(d), n_channels[d-1]
conv(kernel=3, stride=1,padding=1), shape=2**(d),2**(d), n_channels[d]
**batchnorman**
**leakyRelu**
**conv**(kernel=3, stride=1,padding=1), shape=2**(d),2**(d), n_channels[d]
**batchnorman**
**leakyRelu**
**conv**(kernel=3, stride=1,padding=1), shape=2**(d),2**(d), 3
**activation**: tanh

# Supplementary Material

## Supplementary Figure 2. Summary diagram of the methodology

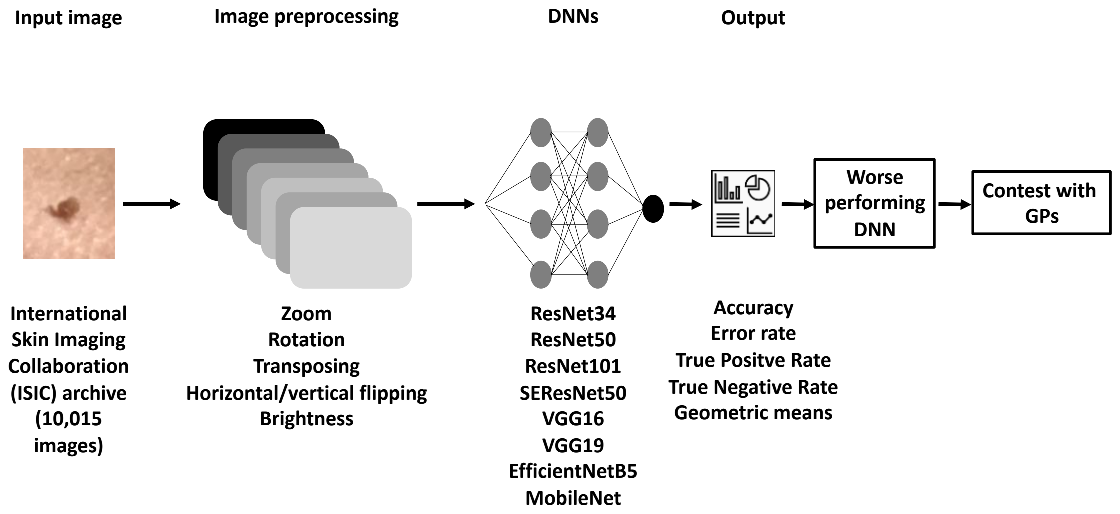


This study used images from the anonymous and annotated HAM10000 dataset publicly available through the International Skin Imaging Collaboration (ISIC) archive [21]. We stochastically split the master set of 10,015 dermoscopic images into training (n=8,313; 83%) and test (N=1,702; 17%) datasets that were completely disjoint. We have evaluated eight different DNNs each characterized by a specific architecture. Because of the variability that exists in the dermatological images analyzed by a particular neural network two steps were taken to reduce overfitting. First, a dropout layer was added and set to 0.5. The second step taken to reduce overfitting was to use data augmentation. These modifications were applied to 66% of the input images. After the model had been trained, a test step was performed in which Sensitivity, specificity, geometric mean, accuracy and error rate were calculated for each dermatological manifestation All metric results were calculated with respect to the class labels documented in the HAM10000 database archive. To compare the accuracy of DNNs with non-dermatologist practitioners, we conducted two different challenges. The first has been aimed to establish the accuracy of general practitioners in classifying images from the HAM10000 dataset without time constraint. The second aimed to determine if physicians could benefit from access to the algorithmic tool during the own classification task.
