# Supplementary material for: Deep Neural Frameworks Improve the Accuracy of General Practitioners in the Classification of Pigmented Skin Lesions
Source: Diagnostics (Basel). 2020 Nov 18;10(11):969. doi: 10.3390/diagnostics10110969 (PMC7698907; doi:10.3390/diagnostics10110969)
Supplement: Supplementary file 1 [file diagnostics-10-00969-s001.zip › New folder/Supplementary Table.pdf]

Supplementary Table S1. Classification metrics for each skin lesion subset of the HAM10000 images using eight different CNNs and low-resolution images without associated clinical features.

CNN	Nev[1]	BK	VL	DTF	ICA	BCC	Mel
ResNet34							
TPR[2]	0.8597	0.4916	0.7307	0.2333	0.3783	0.5384	0.5136
TNR	0.1402	0.5083	0.2692	0.7666	0.6216	0.4615	0.4863
GM	0.6550	0.7578	0.7224	0.7087	0.7486	0.7569	0.7577
ResNet50							
TPR	0.8454	0.5214	0.7000	0.2916	0.3125	0.5151	0.5000
TNR	0.1545	0.4785	0.3000	0.7083	0.6875	0.4848	0.5000
GM	0.6656	0.7575	0.7318	0.7294	0.7352	0.7577	0.7578
ResNet101							
TPR	0.8640	0.4923	0.5121	0.2631	0.3720	0.5384	0.5979
TNR	0.1359	0.5076	0.4878	0.7368	0.6279	0.4615	0.4020
GM	0.6515	0.7578	0.7577	0.7203	0.7476	0.7569	0.7519
SEResNet50							
TPR	0.8934	0.54736	0.6923	0.34615	0.4242	0.6105	0.5302
TNR	0.1065	0.45263	0.3076	0.65384	0.5757	0.3894	0.4697
GM	0.6248	0.75649	0.7339	0.7429	0.7543	0.7503	0.7573
VGG16							
TPR	0.8838	0.5503	0.9411	0.1764	0.3586	0.6734	0.4780
TNR	0.1161	0.4496	0.0588	0.8235	0.6413	0.3265	0.5219
GM	0.6342	0.7563	0.5605	0.6799	0.7453	0.7386	0.7575
VGG19							
TPR	0.8782	0.4555	0.8695	0.2608	0.2631	0.6168	0.4455
TNR	0.1217	0.5444	0.1304	0.7391	0.7368	0.3831	0.5544

GM	0.6394	0.7566	0.6470	0.7194	0.7203	0.7493	0.7560
EfficientNetB5							
TPR	0.8591	0.4776	0.6111	0.2571	0.3380	0.4761	0.5555
TNR	0.1408	0.5223	0.3888	0.7428	0.6619	0.5238	0.4444
GM	0.6554	0.7575	0.7502	0.7181	0.7412	0.7575	0.7559
MobileNet							
TPR	0.9053	0.6117	0.9047	0.3928	0.6122	0.7741	0.6172
TNR	0.0946	0.3882	0.0952	0.6071	0.3877	0.2258	0.3827
GM	0.6117	0.7501	0.6124	0.7507	0.7500	0.7055	0.7493

[1] Nev: melanocytic nevi; BK: bening keratosis; VL: vascular lesions; DTF: dermatofibroma; ICA: intraepithelial carcinoma; BCC: basal cell carcinoma; Mel: melanoma.

[2] TPR: true positive rate; TNR: true negative rate; GM: geometric mean.

Supplementary Table S2. Classification metrics for each skin lesion subset of the HAM10000 images using eight different CNNs and high-resolution images without associated clinical features.

CNN	Nev[1]	BK	VL	DTF	ICA	BCC	Mel
ResNet34							
TPR[2]	0.8615	0.5163	0.7407	0.3437	0.4210	0.5578	0.5234
TNR	0.1384	0.4836	0.2592	0.6562	0.5789	0.4421	0.4765
GM	0.6535	0.7576	0.7189	0.7424	0.7540	0.7558	0.7575
ResNet50							
TPR	0.8761	0.5312	0.7826	0.5555	0.3333	0.5384	0.5555
TNR	0.1238	0.4687	0.2173	0.4444	0.6666	0.4615	0.4444
GM	0.6413	0.7572	0.7017	0.7559	0.7402	0.7569	0.7559
ResNet101							
TPR	0.8659	0.5075	0.5813	0.4285	0.4222	0.5546	0.6060

TNR	0.1340	0.4924	0.4186	0.5714	0.5777	0.4453	0.3939
GM	0.6500	0.7578	0.7537	0.7547	0.7541	0.7560	0.7509
SEResNet50							
TPR	0.8934	0.5625	0.7333	0.4444	0.4705	0.6391	0.5496
TNR	0.1065	0.4375	0.2666	0.5555	0.5294	0.3608	0.4503
GM	0.6248	0.7554	0.7215	0.7559	0.7573	0.7457	0.7563
VGG16							
TPR	0.8839	0.5725	0.9444	0.4000	0.3894	0.6930	0.4972
TNR	0.1160	0.4274	0.0555	0.6000	0.6105	0.3069	0.5027
GM	0.6341	0.7546	0.5546	0.7516	0.7503	0.7337	0.7578
VGG19							
TPR	0.8783	0.4725	0.8846	0.4000	0.4000	0.6422	0.4757
TNR	0.1216	0.5274	0.1153	0.6000	0.6000	0.3577	0.5242
GM	0.6393	0.7574	0.6335	0.7516	0.7516	0.7451	0.7575
EfficientNetB5							
TPR	0.8601	0.5000	0.6315	0.2571	0.3835	0.5098	0.5869
TNR	0.1398	0.5000	0.3684	0.7428	0.6164	0.4901	0.4130
GM	0.6547	0.7578	0.7470	0.7181	0.7494	0.7578	0.7532
MobileNet							
TPR	0.9053	0.6279	0.9200	0.5000	0.6666	0.8000	0.6341
TNR	0.0946	0.3720	0.0800	0.5000	0.3333	0.2000	0.3658
GM	0.6117	0.7476	0.5934	0.7578	0.7402	0.6931	0.7466

[1] Nev: melanocytic nevi; BK: bening keratosis; VL: vascular lesions; DTF: dermatofibroma; ICA: intraepithelial carcinoma; BCC: basal cell carcinoma; Mel: melanoma.

[2] TPR: true positive rate; TNR: true negative rate; GM: geometric mean.

Supplementary Table S3. Classification metrics for each skin lesion subset of the HAM10000 using eight different CNNs and low-resolution images with aggregated clinical data.

CNN	Nev[1]	BK	VL	DTF	ICA	BCC	Mel
ResNet34							
TPR[2]	0.8687	0.4910	0.8260	0.2500	0.6200	0.6200	0.5333
TNR	0.1312	0.5089	0.1739	0.7500	0.3750	0.3750	0.4666
GM	0.6477	0.7578	0.6783	0.7154	0.7481	0.7481	0.7571
ResNet50							
TPR	0.8764	0.5875	0.7500	0.4444	0.4722	0.6206	0.5677
TNR	0.1235	0.4124	0.2500	0.5555	0.5277	0.3793	0.4322
GM	0.6410	0.7531	0.7154	0.7559	0.7573	0.7488	0.7550
ResNet101							
TPR	0.8713	0.5524	0.8500	0.1666	0.2903	0.6179	0.5229
TNR	0.1286	0.4475	0.1500	0.8333	0.7096	0.3820	0.4770
GM	0.6455	0.7561	0.6623	0.6738	0.7291	0.7492	0.7575
SEResNet50							
TPR	0.8887	0.5606	0.7272	0.8571	0.5588	0.7027	0.5449
TNR	0.1112	0.4393	0.2727	0.1428	0.4411	0.2972	0.4550
GM	0.6295	0.7556	0.7235	0.6570	0.7557	0.7311	0.7566
VGG16							
TPR	0.8799	0.5481	1.0000	0.1666	0.5700	0.6372	0.6170
TNR	0.1200	0.4518	0.0000	0.8333	0.4250	0.3627	0.3829
GM	0.6379	0.7564	0.0000	0.6738	0.7544	0.7460	0.7493
VGG19							
TPR	0.8687	0.5325	1.0000	0.3333	0.5000	0.7500	0.5822
TNR	0.1312	0.4674	0.0000	0.6666	0.5000	0.2500	0.4177

GM	0.6477	0.7572	0.0000	0.7402	0.7578	0.7154	0.7537
EfficientNetB5							
TPR	0.8631	0.4468	0.9047	0.5384	0.4186	0.5937	0.4965
TNR	0.1368	0.5531	0.0952	0.4615	0.5813	0.4062	0.5034
GM	0.6523	0.7561	0.6124	0.7569	0.7537	0.7524	0.7578
MobileNet							
TPR	0.8887	0.5606	0.7272	0.8571	0.6315	0.7027	0.5449
TNR	0.1112	0.4393	0.2727	0.1428	0.3684	0.2972	0.4550
GM	0.6295	0.7556	0.7235	0.6570	0.7470	0.7311	0.7566

[1] Nev: melanocytic nevi; BK: bening keratosis; VL: vascular lesions; DTF: dermatofibroma; ICA: intraepithelial carcinoma; BCC: basal cell carcinoma; Mel: melanoma.

[2] TPR: true positive rate; TNR: true negative rate; GM: geometric mean.

Supplementary Table S4. Classification metrics for each skin lesion subset of the HAM10000 using eight different CNNs and high-resolution images with aggregated clinical data.

CNN	Nev[1]	BK	VL	DTF	ICA	BCC	Mel
ResNet34							
TPR[2]	0.8705	0.5088	0.8333	0.6000	0.5263	0.6413	0.5416
TNR	0.1294	0.4911	0.1666	0.4000	0.4736	0.3586	0.4583
GM	0.6461	0.7578	0.6738	0.7516	0.7574	0.7453	0.7568
ResNet50							
TPR	0.9092	0.5977	0.8260	0.2000	0.4406	0.6582	0.5545
TNR	0.0907	0.4022	0.1739	0.8000	0.5593	0.3417	0.4454
GM	0.6071	0.7519	0.6783	0.6931	0.7557	0.7420	0.7560
ResNet101							
TPR	0.8732	0.5683	0.9545	0.6250	0.3636	0.6373	0.5284

TNR	0.1267	0.4316	0.0454	0.3750	0.6363	0.3626	0.4715
GM	0.6439	0.7550	0.5339	0.7481	0.7462	0.7460	0.7573
SEResNet50							
TPR	0.9128	0.6780	0.7307	0.2962	0.2950	0.5066	0.4583
TNR	0.0871	0.3219	0.2692	0.7037	0.7049	0.4933	0.5416
GM	0.6027	0.7375	0.7224	0.7308	0.7304	0.7578	0.7568
VGG16							
TPR	0.8800	0.5654	1.0000	0.6666	0.6279	0.6571	0.6388
TNR	0.1199	0.4345	0.0000	0.3333	0.3720	0.3428	0.3611
GM	0.6378	0.7552	0.0000	0.7402	0.7476	0.7422	0.7457
VGG19							
TPR	0.8746	0.4840	0.9230	0.5714	0.3750	0.6106	0.7903
TNR	0.1253	0.5159	0.0769	0.4285	0.6250	0.3893	0.2096
GM	0.6426	0.7577	0.5891	0.7547	0.7481	0.7502	0.6980
EfficientNetB5							
TPR	0.8640	0.4631	0.9130	0.5384	0.4888	0.6372	0.5167
TNR	0.1359	0.5368	0.0869	0.4615	0.5111	0.3627	0.4832
GM	0.6516	0.7570	0.6025	0.7569	0.7577	0.7460	0.7576
MobileNet							
TPR	0.9040	0.6234	0.9300	0.5321	0.8289	0.7890	0.6764
TNR	0.0959	0.3765	0.0700	0.4678	0.1710	0.2109	0.3235
GM	0.6132	0.7483	0.5790	0.7572	0.6765	0.6986	0.7379

[1] Nev: melanocytic nevi; BK: bening keratosis; VL: vascular lesions; DTF: dermatofibroma; ICA: intraepithelial carcinoma; BCC: basal cell carcinoma; Mel: melanoma.

[2] TPR: true positive rate; TNR: true negative rate; GM: geometric mean.

Supplementary Table S5. Classification metrics for each skin lesion in 163 images of the HAM10000 database by non-dermatologist, general practitioners without access to algorithmic outputs and no time constraint. EfficientNetB5 was used as comparator in the same image dataset.

CNN	Nev[1]	BK	VL	DTF	ICA	BCC	Mel
GPs[2]							
TPR[3]	0.7950	0.2680	0.0200	0.0130	0.0720	0.1780	0.2640
TNR	0.2050	0.7320	0.9800	0.9870	0.9280	0.8220	0.7360
GM	0.6960	0.7220	0.4580	0.4170	0.5830	0.6810	0.7210
EfficientNetB5							
TPR	0.8620	0.6000	0.8620	0.6670	0.5710	0.6000	0.5560
TNR	0.1380	0.4000	0.1380	1.0000	0.4290	0.4000	0.4440
GM	0.6530	0.7520	0.6530	0.9220	0.7550	0.7520	0.7560

[1] Nev: melanocytic nevi; BK: bening keratosis; VL: vascular lesions; DTF: dermatofibroma; ICA: intraepithelial carcinoma; BCC: basal cell carcinoma; Mel: melanoma.

[2] GPs: general practitioners

[3] TPR: true positive rate; TNR: true negative rate; GM: geometric mean.

Supplementary Table S6. Classification metrics for each skin lesion in 70 images of the HAM10000 database by non-dermatologist, general practitioners without (n=35) or with access (n=35) to algorithmic outputs and time constraint of 45 sec/image.

CNN	Nev[1]	BK	VL	DTF	ICA	BCC	Mel
GPs (W/O)[2]							
TPR[3]	0.607	0.142	0.000	0.000	0.033	0.100	0.083
TNR	0.393	0.858	1.000	1.000	0.967	0.900	0.917
GM	0.751	0.656	0.000	0.000	0.503	0.618	0.598

GPs(W)							
TPR	0.686	0.053	0.000	0.000	0.000	0.547	0.356
TNR	0.314	0.947	1.000	1.000	1.000	0.453	0.644
GM	0.736	0.549	0.000	0.000	0.000	0.757	0.745

[1] Nev: melanocytic nevi; BK: bening keratosis; VL: vascular lesions; DTF: dermatofibroma; ICA: intraepithelial carcinoma; BCC: basal cell carcinoma; Mel: melanoma.

[2] GPs (W/O): general practitioners (without); GPs (W): general practitioners (with)

[3] TPR: true positive rate; TNR: true negative rate; GM: geometric mean.
